# Laparoscopic inguinal hernia repair (LIHR): the benefit of the double stitch in the largest single-center experience

**DOI:** 10.1007/s00383-023-05599-4

**Published:** 2023-12-08

**Authors:** Zeni Haveliwala, Simon Eaton, Jayaram Sivaraj, Hemanshoo Thakkar, Sara Omar, Stefano Giuliani, Simon Blackburn, Dhanya Mullassery, Joe Curry, Kate Cross, Paolo De Coppi

**Affiliations:** 1 Great Ormond Street Hospital for Children, London, UK; 2 Surgery Unit, DBC, NIHR Biomedical Research Centre, UCL Great Ormond Street Institute of Child Health, London, UK; 3 Surgery Offices, Zayed Centre for Research, UCL Institute of Child Health, 30 Guilford Street, London, WC1N 1EH UK

**Keywords:** Inguinal hernia, Laparoscopy, Recurrence, Prematurity, Patent processus vaginalis

## Abstract

**Aim:**

To review our experience of laparoscopic inguinal hernia repair (LIHR) regarding complication rates, the practice of closing the asymptomatic patent processes vaginalis (PPV), and comparison of complication rates between pre-term (< 37 week gestation) and term infants.

**Methods:**

Retrospective review of LIHR performed between 2009 and 2021. Repair was performed by intracorporal single or double purse string/purse string + Z-stitch using a non-absorbable suture. Data were analyzed using Chi-squared/Mann–Whitney and are quoted as median (range).

**Results:**

1855 inguinal rings were closed in 1195 patients (943 (79%) male). 1378 rings (74%) were symptomatic. 492 (41%) patients were pre-term. Corrected gestational age at surgery was 55 weeks (31 weeks–14.6 years) and weight 5.9 kg (1–65.5). Closure of contralateral PPV was higher in the premature group (210/397 [53%] vs. 265/613 [43%] *p* = 0.003). There were 23 recurrences in 20 patients, of whom 10 had been born prematurely. The only factor significantly associated with a lower recurrence was use of a second stitch (*p* = 0.011).

**Conclusion:**

This is the largest single-center reported series of LIHR. LIHR is safe at any age, the risk of recurrence is low, and can be corrected by re-laparoscopy. Use of a Z-stitch or second purse string is associated with a significantly lower rate of recurrence.

## Introduction

Inguinal hernia repair is a common procedure performed in pediatric surgical practice. Conventional inguinal hernia repair in children via an open groin approach includes the ligation and division of the hernial sac at the internal inguinal ring. Laparoscopic surgery has been applied in children and involves the closure of the hernia sac with or without any division. While laparoscopy has gained popularity and has been demonstrated to be safe and reproducible in both male and female patients [1–3], the vast majority of the inguinal hernia repairs in children are still performed open [1–3].

At Great Ormond Street Hospital (GOS), we have been performing laparoscopic inguinal hernia repair (LIHR) in pediatric patients as the standard of care (with some exceptions) for the past twelve years. One of the potential advantages of the laparoscopic approach is the ability to assess the contralateral, asymptomatic deep ring, and close it if needed. However, whether an incidentally identified open deep ring subsequently leads to a metachronous inguinal hernia is unclear [4]. Understanding the incidence of incidentally identified open deep rings in pre-term and term infants may clarify whether these need to be closed.

The aims of this study are to review our experience and to identify patient and procedure characteristics of LIHR in pre-term (< 37 weeks gestation) versus term infants, especially the incidence of incidentally identified open deep rings; to calculate the incidence of hernia recurrence in LIHR using one of the largest cohorts of cases in the literature.

## Methods

We performed a retrospective review of a prospectively held database consisting of all LIHR performed at GOS between November 2009 and December 2021. All patients were under 16 years of age. Data were subcategorized into repair performed in pre-term (< 37 weeks gestation) infants versus term (≥ 37 weeks gestation) infants. We collected data on patient demographics, hernia characteristics, and operative outcomes.

### Measured data

Patient demographic data were collected and are presented as median (range). Hernia characteristics were presence of a contralateral patent processus vaginalis (PPV), presence of a concomitant undescended testis on the affected side, and other associated pathology. Primary outcomes were rate of recurrence and incidence of acquired ipsilateral ascending testis. Secondary outcomes were wound complications requiring further intervention.

### Operative details

All cases were performed under general anesthesia by both consultant and trainee operators. Pneumoperitoneum was achieved using an open Hasson technique with a 5 mm port using a curve-linear incision at the umbilicus. A 5 mm 0 degree laparoscope (Karl-Storz, Tuttlingen, Germany) was introduced. Two further 3 mm instruments were introduced via stab incisions in the left and right flanks. Five millimeter instruments were used in selected patients, in whom 3 mm instruments were unsuitable. A Trendelenburg position was used to reduce the contents of the hernia and allow visualization of both rings. The symptomatic PPV was closed with an intra-corporeal purse string using a 3–0 or 4–0 non-absorbable monofilament suture (Prolene, Ethicon, Johnson & Johnson, Raritan, NJ US) and an intra-corporeal knot. The purse string was reinforced with an overlying “Z” stitch, or another purse string based on surgeon preference. If a contralateral PPV was identified (confirmed by probing with the laparoscopic instrument), this was closed with a purse string with or without an overlying “Z” stitch, based on surgeon preference. The fascia at the umbilical port-site was closed with a suitable 2–0 suture, while the side entrances were closed using an absorbable 4–0 or 5–0 suture and skin glue. Patients < 60 weeks corrected gestational age were discharged following surgery on the same day if they met discharge criteria. Undescended testes were noted but concurrent orchidopexy was not routinely performed.

### Statistical analysis

Chi-squared test and Mann–Whitney tests were used for comparison. Binary logistic regression analysis was also performed using SPSS v. 27. *p* value < 0.05 was considered significant.

The study was registered with the hospital audit committee.

## Results

One thousand one hundred and ninety-five patients (944 male, 79%) underwent laparoscopic repair of an inguinal hernia in the 12-year period. Six hundred and forty-six patients had a symptomatic right-sided hernia, 365 left and 183 were bilateral. One patient was diagnosed with an inguinal hernia incidentally during laparoscopy for an alternative pathology.

Median patient age at operation was 55 weeks (31 weeks to 14.6 years). Median weight was 5.5 kg (1–65.5 kg). The youngest patient was 1 day old. The distribution of age at operation is categorized in Table 1; 46 patients were operated at a corrected gestational age of less than 40 weeks.

**Table 1:** Distribution of age at operation in our cohort

Age (years)	Number of patients
< 1	950
1–2	96
> 2	149

One thousand and ten patients (84%) underwent an elective procedure. Seventy-nine (7%) required emergency surgery for an incarcerated hernia.

### Hernia characteristics

#### Contralateral PPV ligation

One thousand eight hundred and fifty-five inguinal hernia rings were closed, of which 1378 (74%) were symptomatic. An asymptomatic contralateral PPV was found in 474 (40%) patients. Although a contralateral PPV was more common in children with symptomatic left inguinal hernias (178/364; 49%) when compared to children with symptomatic right-sided hernias (296/646; 46%) this was not statistically significant (*p* = 0.3). One patient (0.08%) had a metachronous inguinal hernia after false-negative assessment of a closed contralateral PPV at initial hernia repair. A contralateral PPV was significantly more likely in males (OR 1.7, 95% CI 1.3–2.4, *p* = 0.001) and in patients with a lower corrected gestational age (OR 1.01, 95% CI 1.00–1.02, *p* = 0.003 per month). There were no metachronous hernias following asymptomatic PPV closure.

#### Concomitant undescended testis

Thirty-one testes were undescended in 25 patients at initial hernia repair. One testis descended on follow-up and did not require a formal orchidopexy. Two testes atrophied, both of which had presented with an incarcerated hernia on the affected side. Twenty-eight testes (90%) that were undescended prior to hernia repair underwent subsequent orchidopexy. Two of these were intra-abdominal at the time of hernia repair, of which one underwent concurrent first-stage Fowler–Stephens orchiopexy.

#### Associated pathology

One female patient who presented with an incarcerated right inguinal hernia was found to have bilateral hernias and complete androgen insensitivity syndrome.

### Operative outcomes

#### Recurrence

**Fig. 1:**
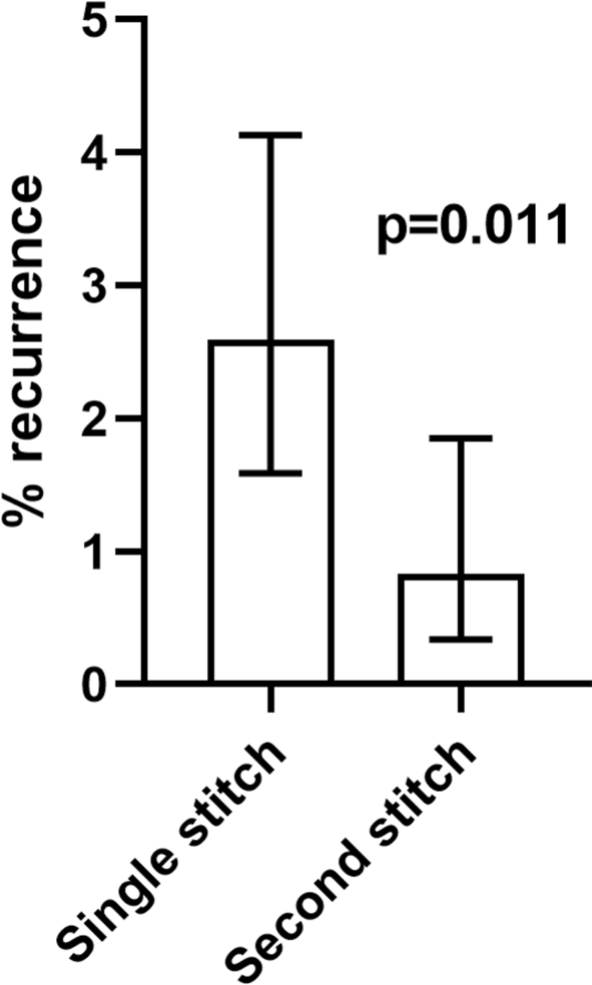
Box and whisker plot for recurrence rate by hernia depending on whether a single stitch or second stitch (Z-stitch or second purse string) was used. Data are percent with 95% confidence intervals and demonstrates a significantly lower recurrence rate with the use of a second stitch (*p* = 0.011)

Twenty patients (1.7%) had a recurrence, including 3 bilateral recurrences, giving a recurrence rate of 1.7% (23/1378) per symptomatic hernia repaired, or 1.2% per patent ring closed. Recurrences in eight patients (40%) were treated laparoscopically. One patient had significant co-morbidities and died before their re-do procedure. Univariate analysis of factors associated with hernia recurrence (Table 2) showed that recurrence was not associated with elective/expedited/emergency surgery, whether the operation was performed by trainee or consultant, or suture material. Neither corrected gestational age nor weight at operation was significantly different. The only factor significantly associated with a lower rate of recurrent hernia was use of a Z-stitch (Fig. 1); recurrence was significantly lower in hernias where a Z-stitch had been used (*p* = 0.028) or where any second stitch (Z or second purse string) was used (*p* = 0.011).

**Table 2:** Univariate analysis of factors associated with recurrence

	Recurrence	No recurrence	*p* value
Urgency of operation			
Elective	19	1157	0.14
Expedited	4	109	
Emergency	0	86	
Operating surgeon			
Trainee	12	554	0.39
Consultant	10	758	
Both	1	28	
Purse string			
Yes	23	1332	0.77
No	0	5	
Second purse string			
Yes	1	124	0.41
No	22	1201	
Z-stitch			
Yes	5	592	**0.028**
No	18	734	
Z-stitch or second purse string			
Yes	6	715	**0.011**
No	17	640	
Corrected age at repair (weeks)	54 (36–294)	55 (31–761)	0.86
Weight at repair (kg)	5.3 (2.3–15.4)	5.8 (1–66)	0.52

Items highlighted in bold demonstrate statistical significance

Binary logistic regression analysis similarly indicated that either Z-stitch alone (*p* = 0.038), or second purse string/Z-stitch (*p* = 0.031) were the only factors significantly associated with prevention of recurrence. Z-stitch or second purse string were used more frequently later in the series, so it was difficult to independently assess whether there was a learning curve effect, although analysis of recurrence in those patients not having a second stitch suggested that there was no association between learning curve and recurrence (*p* = 0.34).

#### Acquired undescended testis

Twelve testes in ten patients required subsequent surgery for iatrogenic cryptorchidism after inguinal hernia repair. The need for formal orchidopexy after inguinal hernia repair was not affected by gestational age at birth. Four ascending testes occurred following repair of an asymptomatic contralateral PPV.

#### Other

Three patients had an unplanned return to theater to control bleeding from port sites. There were no specific clotting abnormalities identified to explain this complication. No patients had significant bleeding requiring transfusion. There were no iatrogenic complications (bladder perforation or transection of vas deferens) from laparoscopic repair.

#### Follow-up

Patient data on follow-up were analyzed until April 2022.

### Pre-term versus term analysis

Demographic data were compared (Table 3). Four hundred and ninety-two pre-term (< 37 week gestation) patients underwent inguinal hernia repair in our cohort, with a significantly greater proportion of males in the pre-term group. There was also a significantly different distribution of sidedness between pre-terms and terms, with relatively more right-sided hernias in those born at term. A contralateral PPV requiring treatment was seen in 209. Finding of a contralateral PPV requiring treatment was significantly increased (*p* = 0.003) when compared to laparoscopy findings in term (≥ 37-week gestation) infants. Pre-term patients did not have increased complications; undescended testis, recurrence, port-site hernia or bleeding.

**Table 3:** Demographic data of pre-term and term cohort

	Pre-term (< 37 weeks gestation at birth), *n* = 492 (%)	Term (> 37 weeks gestation at birth), *n* = 701 (%)	*p* value
Demographic data			
Gender			
Male	414 (84%)	529 (75%)	< 0.0005
Female	78 (16%)	172 (25%)	
Symptomatic hernia			
Right	217 (44%)	429 (61%)	< 0.0005
Left	180 (37%)	184 (26%)	
Bilateral	94 (19%)	88 (13%)	
Incidental	1	0	
Weight at operation	4.7 kg (1–23.8)	6.7 kg (1.74–65.5)	< 0.0005
Corrected gestational age	11.5 (7.2–101) months	13.7 (8.6–176) months	< 0.0005
Hernia characteristics			
Asymptomatic PPV	209/397 (52%)	265/613 (43%)	**0.003**
Operative outcomes			
Recurrence	10	10	0.51
Acquired undescended testes	6/414 (1.4%)	4/529 (0.7%)	0.30

Item highlighted in bold demonstrates statistical significance

## Discussion

Inguinal hernia repair is one of the most common procedures performed by a pediatric surgeon, with a reported incidence of 0.8–4.4% [5]. Failure of closure of the processus vaginalis, leads to a persistent canal that allows abdominal contents to protrude through the deep ring. The persistence of the canal carries a risk of incarceration and strangulation of the contents, therefore surgical intervention is indicated in all.

Laparoscopic hernia repair was first described by El-Gohary [1]. It was initially used as an option for inguinal hernia repair in female patients due to concerns about damage to the vas and vessels in males. This was followed by laparoscopic repair in males by Esposito [2] who initially described laparoscopy to treat recurrence following open hernia surgery. Subsequently, laparoscopic repair has become one of the standard approaches to treat inguinal hernias in children, and was adopted by Great Ormond Street Hospital in 2009. A prospectively held database of all patients undergoing the procedure has been maintained since. Laparoscopic repair carries with it several advantages including; improved post-operative pain and cosmesis, faster operative times for bilateral cases, identification of metachronous hernia and less trauma to delicate cord structures [6]. Moreover, in cases of incarceration, the laparoscopic technique appears safer and easier to perform than open repair and can be done without the need to wait after reduction [7].

When discussing minimally invasive surgery and inguinal hernia repair, it is important to firstly recognize that a wide range of techniques exist. These can broadly be divided into intra-corporeal or extra-corporeal depending on the method by which the needle is inserted into the peritoneal cavity and how the deep ring is closed [8]. Our operative approach is similar to that described by Montupet [9]. Most clinicians, in the present series, chose to place a second purse string or Z-stitch [10] on the symptomatic side/s. Treatment of the asymptomatic PPV was with a single purse-string suture only with or without an overlying “Z” stitch.

Controversy exists regarding the assessment and treatment of the asymptomatic (PPV); however, closure is generally recommended to reduce the risk of a metachronous hernia. A recent evidence-based guideline on minimal access approaches to inguinal hernia, published by the International Pediatric Endosurgery Group in 2016, found the incidence of contralateral PPV is 20–30% [6]. In our series, the incidence of asymptomatic PPV was 40%. It was more likely in children presenting with symptomatic left sided hernias, when compared with right, as expected from the embryological descent of the testis and delayed closure of the processus vaginalis on the right [11]. The natural history of the PPV is one of the spontaneous closures.

The incidence of PPV and inguinal hernia in a full-term infant is between 3 and 5%, which increases up to 30% in pre-term infants [12, 13]. Pre-term infants are also considered to be at increased risk of metachronous inguinal hernia [14]. A study by Burgmeier et al. [15] reviewed the findings of a contralateral PPV in term versus pre-term infants, which demonstrated a contralateral PPV rate in the pre-term group of up to 58.8%. This finding is supported by our study, which also demonstrates a significantly higher incidence of contralateral PPV (52%) in pre-term infants. There is no evidence to determine if a PPV seen at laparoscopy will become symptomatic but the risk of a contralateral asymptomatic PPV developing into a metachronous hernia is approximately 25% [6]. Laparoscopic assessment is easy, feasible and closure of the PPV can potentially prevent morbidity associated with a metachronous hernia. It seems to be more important in the pre-term infants, where the risk of a contralateral PPV is above 50%.

We note that laparoscopic assessment of the contralateral PPV is not failsafe. A meta-analysis by Zhong et al. [16] demonstrated 1.31% rate of metachronous hernia development in children with a false-negative finding of a contralateral PPV during laparoscopy. The incidence of false-negative closed ring in our series was 0.08%, with one patient in our cohort developed a metachronous inguinal hernia following laparoscopic inguinal hernia repair, where initial assessment had not demonstrated a contralateral PPV. Overall, the assessment and management of a contralateral PPV are recommended during laparoscopic inguinal hernia repair.

The reported disadvantages of laparoscopic inguinal hernia repair are a potentially higher recurrence rate. The reported recurrence rate after laparoscopic hernia repair is between 0% and 5.5% (mean 1.4%) [3]. Age and weight have previously been reviewed as indicators for recurrence in LIHR [17]. Our cohort of patients included patients from day 1 of life up to 14 years at operation, and these factors did not have an impact on recurrence. Comparing laparoscopic and open approaches, the recurrence rate in a systematic review and meta-analysis on by Bada-Bosch et al. [18] demonstrated no significant differences. Higher recurrence rates (up to 5.5%) quoted in the systematic review by Esposito [3] on LIHR are due to the inclusion of a paper by Koivusalo [19] in which one recurrence was seen after laparoscopic hernia repair in a small cohort (18 patients). This leads to an overestimation of the true population recurrence rate. The recurrence rate in our cohort of 1195 patients was 1.6%. However, this rate reduced to 0.8% when an intra-corporeal second Z-suture stitch was used on the symptomatic side. The use of a Z-stitch was first described by Schier, initially in girls [20], and then in boys [10]. Following early experience with LIHR in our center, a double stitch/Z-stitch was adopted, which resulted in a significant reduction in the rate of recurrence. This paper provides a set of data in which to accurately assess the recurrence rate, in a large cohort of patients with follow-up of up to 12 years [6].

Iatrogenic cryptorchidism is a recognized complication following inguinal hernia repair, which is postulated to occur as a result of adhesions created by dissection of the processus vaginalis (PV), leading to scarring of the cord [21]. Further growth of the pediatric patient can result in a descended testis ascending over time. The laparoscopic approach, in theory, would cause less scarring as there is no disruption to the processus vaginalis. However, persistence of the PV may, in part, impact the rate of testicular ascent. 90% of patients in our cohort with undescended testis pre-herniotomy required a formal orchidopexy. The majority of these were in patients less than 6 months of age at the time of hernia repair. Although spontaneous descent of undescended testis in children < 6 months is reported [22], it is not common. A large proportion of our cohort were born prematurely, which also influences the prevalence of undescended testis. It is not possible to determine if the testes that were undescended prior to LIHR would have undergone spontaneous descent. Twelve testes ascended following LIHR in our cohort. This number may reflect the lack of documentation of pre-operative testicular position, as well as the potential impact of the persistence of the PV. Overall, LIHR is associated with less complications when compared to open repair, with specific reference to the incidence of iatrogenic cryptorchidism [23].

Limitations of this study include its retrospective design resulting in an heterogenous protocol for technique and patient characteristics. However, our statistical analysis suggests differences in technique did not alter the rate of recurrence. The practice of documentation of post-operative testicular position is not standardized; therefore, the observation of iatrogenic cryptorchidism may be overestimated. We also acknowledge that some patients may have developed recurrence that did not present to GOS and, therefore, alter our incidence of recurrence.

## Conclusions

In conclusion, this is one of the largest single-center experiences on the laparoscopic repair of inguinal hernia in children. This technique can be easily performed in a range of pediatric patients ranging from 1 to 66 kg, showing a recurrence rate that is comparable to what has been published for open repair, particularly when a second suture is used. While contralateral PPV closure in our cohort was performed in 40%, laparoscopic repair has the advantage to allow visualization and closure, which may be particularly relevant in neonates and infants.

## Data Availability

The data that support the findings of this study are available on request from the corresponding author, PDC.
